# Comparative Liquid Chromatographic Study for Concurrent Determination of Canagliflozin and Metformin in Combined Tablets

**DOI:** 10.1155/2017/9197230

**Published:** 2017-12-03

**Authors:** Wafaa A. Zaghary, Shereen Mowaka, Moataz S. Hendy

**Affiliations:** ^1^Pharmaceutical Chemistry Department, Faculty of Pharmacy, Helwan University, Ein Helwan, Cairo 11795, Egypt; ^2^Pharmaceutical Chemistry Department, Faculty of Pharmacy, The British University in Egypt, El-Sherouk City, Cairo 11837, Egypt; ^3^Analytical Chemistry Department, Faculty of Pharmacy, Helwan University, Ein Helwan, Cairo 11795, Egypt; ^4^The Center for Drug Research and Development (CDRD), Faculty of Pharmacy, The British University in Egypt, El-Sherouk City, Cairo 11837, Egypt

## Abstract

New HPLC-UV method (method A), for simultaneous determination of metformin (MET) and canagliflozin (CANA), was developed and compared to another novel UPLC-UV method (method B) in their tablet combination. Concerning method A, isocratic separation was done by C18 column (100 mm × 2.1 mm, 3 *μ*m) using methanol and 0.03 M phosphate buffer (75 : 25, *v*/*v*) at pH 3.2 as a mobile phase. Meanwhile, chromatographic separation in method B was achieved via Hypersil® gold (50 mm × 2.1 mm, 1.9 *μ*m). Mobile phase was methanol and 0.03 M phosphate buffer at ratio of 80 : 20  *v*/*v*. In both, detection was done at wavelength of 240 nm. Method A showed satisfactory linearity results over 1–50 *μ*g·mL^−1^ and 0.5–100 *μ*g·mL^−1^, while method B linearity was at 0.1–50 *μ*g·mL^−1^ and 0.25–100 *μ*g·mL^−1^ for CANA and MET, respectively. In terms of accuracy and precision, method A accuracy was 99.81 ± 0.73 and 99.37 ± 0.54, while method B gave accuracy of 99.47 ± 1.03 and 99.73 ± 0.89 for CANA and MET, respectively. For precision, the % RSD was found to be less than 2% for three concentrations analyzed three times. The two methods are convenient for quality laboratories, yet the UPLC method offered the advantage of shorter run times and higher sensitivity.

## 1. Introduction

Diabetes mellitus type 2 (DMT2) is a progressive disease, and to fully control hyperglycemia after initial monotherapy and lifestyle modification, combination treatments are usually needed to maintain glycemic control [1].

**Figure 1:**
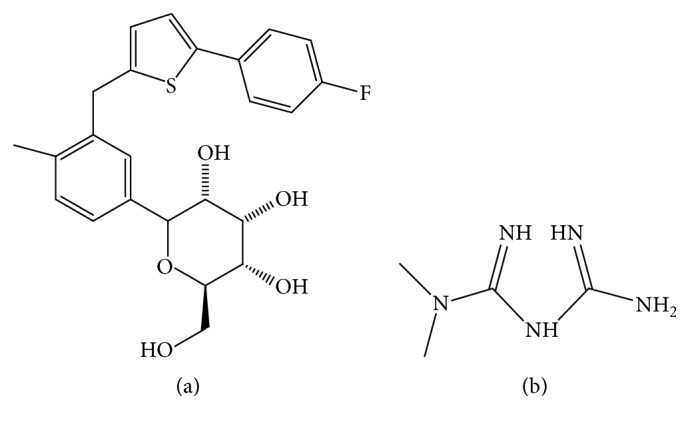
Structures of canagliflozin (a) and metformin (b).

Metformin hydrochloride (MET) (Figure 1(b)) is the most widely used drug for the treatment of DMT2 with a dimethylbiguanidine structure. Metformin-based combinations are found to be better in efficacy and tolerance rather than other single hypoglycemic agent therapy. The therapeutic properties of metformin are due to its action in suppressing glucose production by liver, especially gluconeogenesis. Also metformin increases the insulin sensitivity in peripheral tissue [2, 3].

Canagliflozin (CANA), the other agent in this binary study (Figure 1(a)), is sodium glucose cotransporter 2 (SGLT2) inhibitor, which was developed for DMT2 treatment by Janssen Pharmaceuticals Inc. 2014. Canagliflozin improves urinary glucose excretion via decreasing the glucose renal threshold. SGLT2 is glucose transporter with low affinity and high capacity which is the main cause for renal reabsorption of glucose [4].

Because CANA is a relatively new drug, there are no official methods for its analysis in pharmacopeias but MET has authentic techniques of analysis in the British Pharmacopeia (BP) [5] and United State Pharmacopeia (USP) [6]. Referring to the literature, sparse methods were proclaimed [7–9] for concentration assurance of CANA and MET simultaneously in pharmaceutical preparations or in laboratory prepared mixtures. An objective comparison between the reported and proposed methods was manifested in Table 1 to facilitate proper understanding of the aim of this study.

Other few methods were found for determination of CANA alone, either in dosage form or in plasma. Patel et al. [10] and Kaur et al. [11] achieved concentration determination of CANA in bulk and in its dosage form using UV-spectroscopy. While Suneetha and Sharmila [12] achieved more sensitive method using reversed phase HPLC technique, Iqbal et al. [13, 14] have performed UHPLC method for detection of CANA in plasma using MS/MS and fluorescence detectors each in a separate study. Despite the low limit of detection and quantification achieved in this study, mass and fluorescence detectors are considered quiet expensive and less notorious. They may not be used as regular method in quality control laboratories.

On the contrary, various analysis methodologies were developed for investigation of MET using both spectrophotometry and chromatographic techniques. Metformin was determined via UV-spectrophotometry in presence of the recent empagliflozin in their dosage form [15] and of trending gliptins: alogliptin and linagliptin either in prepared mixtures or in marketed dosage forms [16, 17]. In addition to that, there are remarked methods reported for analysis of MET utilizing either ultra or high performance liquid chromatography with other hypoglycemic agents such as empagliflozin [18], linagliptin, alogliptin, pioglitazone [19], sitagliptin [20, 21], and sitagliptin degradation product. Recently, the sensitive LC-MS technique with various bioanalytical applications was also found in literature for MET detection and quantification [22–27].

There is no UPLC-UV method found, for CANA and MET simultaneous quantification in tablets. Moreover, UPLC is proven to be more economic than HPLC with less organic solvent consumption and less run time and resulting in relatively more sharp peaks. Besides, UPLC offers higher sensitivity in nanogram level facilitating development of new bioanalytical methods for the studied drugs.

This work aims to evolve new, yet more sensitive UPLC-UV and HPLC-UV methods for concurrent quantification of MET and CANA in bulk and in their pharmaceutical preparation. This is followed by full validation for each method. The authors' idea for a comparative study using two different stationary phases has emerged from the need to find the most suitable analytical method in short time to be applicable in QC labs giving the analyst the choice of the preferable detector according to the underlying application. Furthermore, outputs from a comparative study using two columns with different dimensions will be useful for analysts working in the area of drug quality control.

## 2. Experimental

### 2.1. Instrumentation for Methods A and B

The wielded liquid chromatography facility was a UPLC of Thermo Fisher with Ultimate 3000 Ultra Performance Liquid Chromatography (USA). UV Detector (DAD, 3000 RS) and (WPS-3000TRS, Thermo Scientific) (USA) autosampler were employed. For HPLC technique (method A) a BDS Hypersil (USA) C18 column (100 × 3 mm, 3 *µ*m) was used and for UPLC technique (method B) Hypersil gold (USA) C18 column (50 mm × 2.1 mm, 1.9 *μ*m) was used. Software utilized for LC system was ChromLeon 7. Solvent degassing was utilized using Elmasonic S 60 H water bath sonicator (Germany). To adjust pH, Jenway digital pH meter (UK) was employed.

### 2.2. Source of Samples and Reagents

Industrial grade row material of CANA and MET and Invokamet® tablets that contain in each tablet 50 mg CANA and 1000 mg MET were obtained by Janssen Pharmaceuticals, Inc. Co. (USA). Metformin (MET) was kindly supplied by “Chemical Industries Development” (CID) Cairo, Egypt; canagliflozin (CANA) was purchased from Baoji Guokang Bio-Technology Co., Ltd., China. CANA and MET bulk purity were found to be 99.90% and 99.88%, respectively. Deionized water was produced in-house, and acetonitrile, methanol, and potassium phosphate monobasic powder of HPLC grade were purchased from Sigma Aldrich.

### 2.3. Chromatography Separation Conditions

#### 2.3.1. High Performance LC Separation (Method A)

Chromatographic method was applied via Hypersil BDS C18 column (100 × 3 mm, 3 *µ*m) from Thermo Scientific, New York, USA. A binary mobile phase consisting of 75% methanol and 25% (0.03 M) phosphate buffer pH maintained at 3.2 adjusted by phosphoric acid was used. Prior to mobile phase usage, it was Millipore-filtered through 0.2 Millipore filter by vacuum Buchner and then degassed. The separation was done at 240 nm. Mobile phase was pumped in 1.3 mLmin^−1^ flow rate, where isocratic elution was done at room temperature. 10 *μ*L was adapted as sufficient injection volume.

#### 2.3.2. Ultra Performance LC (Method B)

Separation was carried out by a Hypersil gold column (column packed with ultrapure silica), 50 × 2.1 mm (1.9 *μ*m). Isocratic elution was utilized, and mobile phase was prepared as a mixture of methanol and 0.03 phosphate buffer (80 : 20, v/v). pH was adjusted at 3.5 using phosphoric acid. 240 nm wavelength was selected. Mobile phase was filtered initially using 0.2 *μ*m membrane filter and then degassed before its use. Flow rate of 0.4 mLmin^−1^ was adapted. Separation was done at room temperature with 10 *μ*L injection volume.

### 2.4. Stock Preparations

CANA and MET stock solutions of concentration 1 mg·mL^−1^ were initially accurately prepared with methanol. By diluting the corresponding stock solutions with mobile phases, we obtain the working solutions that were used in each method (A and B). All of them were conserved at 4°C and junked after 30 days.

### 2.5. Preparation of Tablet Samples

Ten tablets of Invokamet 50 CANA/1000 MET were solely weighed, powdered, and blended. Specific quantity of the obtained powder contains 100 mg of MET and 5 mg of CANA was dissolved in 30 mL of methanol in a 100 mL volumetric flask, sonicated for 20 min. The solution was filtered and made up to the final volume with methanol. Then certain dilutions were done to prepare the investigated concentrations of the two components via mobile phase. This step was accompanied with addition of precise calculated amount of pure drug to achieve standard addition technique.

### 2.6. Applied Procedures

#### 2.6.1. HPLC Method Calibration Curve

Calibration curves were built up via preparing five solutions of each drug using the mentioned mobile phase within ranges of concentration: 1–50 *µ*g·mL^−1^ and 0.5–100 *µ*g·mL^−1^ for CANA and MET, respectively. 10 *μ*L aliquots were injected onto the stationary phase. Calibration curves were then built up for each drug by plotting the peak area of each drug absorbance against its corresponding concentrations.

#### 2.6.2. UPLC Method Calibration Curve

Calibration curves were built up by preparing five solutions of each drug using the mobile phase in ranges of concentration of 0.1–50 *µ*g·mL^−1^ and 0.25–100 *µ*g·mL^−1^ for CANA and MET, respectively, and then only 10 *μ*L was injected. By plotting area under the peak (AUP) of the corresponding drug against its concentration, the calibration curve was attained.

#### 2.6.3. Study of CANA and MET in Laboratory Mixtures and Invokamet Tablets

The procedures declared earlier were applied for different ratios of CANA and MET and for tablet samples, prepared under Section 2.5. Different concentrations of the investigated drugs were obtained from each computed equation of regression.

## 3. Results and Discussion

It is worth mentioning that the proposed methods are the first methods applying UPLC techniques for the analysis of the studied drugs rather than HPLC technique. UPLC is more advantageous [28] in terms of withstanding the high system back pressure, improved resolution, shorter run times, and fewer consumables. Also the proposed methods were developed with simple mobile phase which enables their further application on different detectors. The obtained results confirmed the validity of the methods showing better resolution and sharp peaks.

Using a Hypersil gold C18 column (50 × 2.1 mm, 1.9 *μ*m) showed better results than the commonly used Symmetry® C18 column (100 × 2.1 mm, 3 *μ*m). When high pressure applied in UPLC was combined with 1.9 *μ*m particles, high peak capacity was observed which is crucial for sensitive CANA determination due to its low contribution in the pharmaceutical combination with MET.

### 3.1. Chromatographic Investigations concerning Separation Conditions

**Figure 2:**
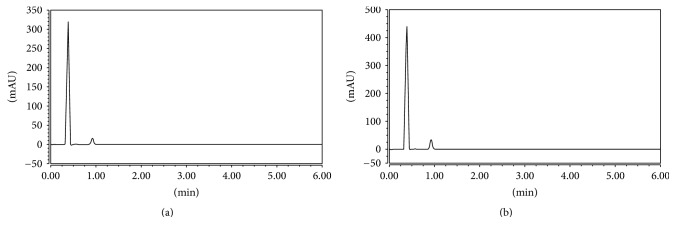
(a) HPLC chromatogram of laboratory mixture containing 50 *μ*g·mL^−1^ of MET at 0.4 min and 25 *μ*g·mL^−1^ of CANA at 0.9 min. (b) UPLC chromatogram of a laboratory mixture containing 50 *μ*g·mL^−1^ of MET at 0.4 min and 25 *μ*g·mL^−1^ of CANA at 0.9 min.

**Figure 3:**
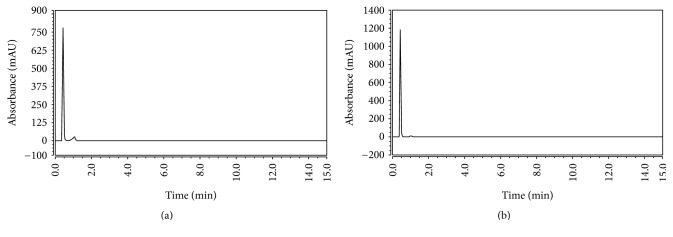
(a) HPLC chromatogram of Invokamet tablet extract containing 60 *μ*gmL^−1^ of MET at 0.4 min and 3 *μ*gmL^−1^ of CANA at 0.9 min. (b) UPLC chromatogram of Invokamet tablet extract containing 60 *μ*gmL^−1^ of MET at 0.4 min and 3 *μ*gmL^−1^ of CANA at 0.9 min.

Concerning method A, HPLC analysis of the drugs was firstly tried using various combinations of either methanol or acetonitrile with water, yet no separation occurred. Addition of buffer was then decided. Phosphate buffer was chosen with two concentrations (0.03 and 0.05 M) with varying ratios with the organic phase. All reported methods showed separation using acetonitrile. So it was implemented first with diverse ratios (from 40% to 75%) of the total mobile phase. Separation was done using acetonitrile and phosphate buffer; methanol then was tried to separate the mixture owing to its lower cost relatively to acetonitrile. The ratio of 75 : 25 methanol : 0.03 M phosphate buffer* v/v* (pH: 3.2) gave the best peak resolution with valid, satisfactory, and rational determination values as revealed in Figures 2 and 3. Mobile phase was pumped on HPLC BDS Hypersil C18 column (100 × 3 mm, 3 *µ*m). Flow rate was optimized to give each peak a smart outline with optimum retention time; it was adjusted to be 1.3 mL·min^−1^. Separation was done at wavelength of 240 nm with no applied temperature on column.

Targeting the optimum detection of the studied drugs, method B was applied using an UPLC Hypersil gold column (50 × 3 mm, 1.9 *µ*m). Mobile phase of methanol or acetonitrile with water results in no separation. Phosphate buffer (0.03 M) was tried in increasing ratios till 20% of the total mobile phase with methanol pumped into the stationary phase implementing isocratic mode; this achieves separation with smart peak shapes. Certainly, optimization of the mobile phase pH was a must. It was expected to be in the acidic region (2.5 and 3.5) to ensure its value below the pka of the studied drugs by more than two units; therefore adjusting pH to 3.5 using phosphoric acid showed the best results. Wavelength of detection was also 240 nm for achieving highest sensitivity for two drugs. In addition, the methanol percent was essential for this technique to improve the resolution between the two eluted peaks obtained with improved shape and smooth outline in room temperature. 0.4 mL·min^−1^ was the flow rate and injection volume was 10 *μ*L.

### 3.2. Validation of the Methods

Validation was done in accordance with ICH guidelines [29].

#### 3.2.1. Linearity

Under the optimum conditions, CANA and MET were evaluated by studying certain different concentrations of each drug. Satisfactory linearity was attained between area under the curve (AUC) and its corresponding concentration of each drug; equations of regression were obtained with high value of correlation coefficient as listed in Table 2. Method A showed satisfactory linearity within concentration ranges of 1–50 *µ*g·mL^−1^ and 0.5–100 *µ*g·mL^−1^ for CANA and MET, respectively, while method B showed good results using ranges of 0.1–50 *µ*g·mL^−1^ and 0.25–100 *µ*g·mL^−1^ for CANA and MET, respectively.

#### 3.2.2. Accuracy and Precision

Results' accuracy was verified through calculation of percentage recovery of five concentrations of each drug by each method (A and B). Besides, calculation of percentage recovery of each drug in laboratory prepared mixture was carried out. The findings include standard deviations and the mean of the recovery is mentioned in Table 2. Precision was verified by analyzing three concentrations of both CANA and MET by each method three times, within the same day to test intraday repeatability. Then to test interday precision and to confirm reproducibility, analysis of three diverse concentrations of the analytes was done on three successive days using the procedures declared earlier. The resultant % RSD was found to be less than 2% in the three concentrations, as found in Table 2.

#### 3.2.3. Robustness

Robustness of methods A and B was verified by the peak area uniformity of the analytes after intentional minor changes performed in chromatographic conditions. This was ascertained by studying the effect of minor changes in experimental parameters on the resolution between the two peaks of MET and CANA. For the HPLC method using the 3-micron C18 column, flow rate was altered from 1.3 mLmin^−1^ to 1.2 mLmin^−1^ and 1.4 mLmin^−1^, the ratio of the organic solvent was altered by %  ±1, and the value of pH of the mobile phase was changed from 3.2 to 2.9 and 3.1; there was no significant change in the results, indicating good robustness of those methods.

#### 3.2.4. Specificity

In the present work, specificity was tested by analyzing four diverse concentrations of each drug using methods A and B in the presence of other drugs as in laboratory prepared mixtures (Table 3) and in presence of additives and excipients of the combination dosage form as represented in Table 4.

#### 3.2.5. Limit of Detection and Limit of Quantification for the Proposed Methods

Limit of detection (LOD) and limit of quantification (LOQ) were computed and represented in Table 2. LOD is the concentration of any analyte at signal/noise ratio of 3.3; meanwhile LOQ is at signal/noise ratio of 10.

#### 3.2.6. System Suitability Tests

Those tests were done to ensure that the chromatographic method is sufficiently reproducible, which mainly include column efficiency, tailing factor of chromatographic peaks, and resolution between peaks. All these results are found in Table 5.

#### 3.2.7. Standard Addition Technique Application on Dosage Forms

This chromatographic methods were satisfyingly utilized for analysis of a pharmaceutical dosage form (Figure 2). Moreover, standard addition technique was employed. This technique is done by adding different known amounts of the pure drug to different known concentrations previously prepared, of the drug product using the procedure mentioned above. The final concentrations were then estimated using each corresponding equation of regression; the date is represented in Table 4.

### 3.3. Statistical Analysis

The obtained results for analysis of CANA and MET by those investigated UPLC and HPLC methods were statistically compared to the reported methods for CANA [7] and MET [27]. The values of computed *t* and *F* were lower than their tabulated values at 95% confidence level. This uncovers that there was no momentous difference concerning precision and accuracy as presented in Table 6.

### 3.4. Remarks about the Procedure

The lack of UPLC-UV methods for concurrent analysis of CANA and MET in tablets has motivated us to propose and develop such a method (method B). Based on our planned future work, this comparative study is considered a crucial step to decide which method can be further employed for the analysis and quantification of the drugs studied, with their different degradation products or in biological fluids. Furthermore, as in Table 1, the proposed HPLC method (method A) has many advantages over the previously reported HPLC-UV methods including the usage of a simpler mobile phase with lowest buffer content, shorter run time, studying the validation parameters, and taking in consideration the ratio of two components in their pharmaceutical preparation.

Moreover, the developed UPLC technique (method B) has other major outcomes that include detection at most sensitive wavelength for the two studied drugs after preliminary investigation, using simple mobile phase which is a mixture containing higher ratio of organic phase with lowest concentration of phosphate buffer (0.03 M), better resolution between peaks, adjusted pH less than the pka of the considered drugs by more than two units, lower LOD and LOQ values, and high throughput analysis, not to mention the sensitivity of the method which was able to detect CANA and MET in a concentration range between 100 ng·mL^−1^ and 50000 ng·mL^−1^ and 250 ng·mL^−1^ and 100000 ng·mL^−1^, respectively.

## 4. Conclusion

The proposed comparative chromatographic study has revealed that UPLC-UV (method B) is more sensitive. Moreover, not only does it offer more advantages, elegant complete separation, but also, compared to the HPLC-UV method, it offers shorter time of development and efficient analysis. Linearity at nanogram range using method B has supported our future plans for the analysis of the same drugs in the presence of their degradation products and in biological fluids. Furthermore, any of the proposed methods is suitable to be used conveniently in quality control laboratories.

## Figures and Tables

**Table 1:** Comparison between different LC-UV reported methods and the proposed methods for simultaneous determination of CANA and MET.

Methods	Column	Mobile phase	Run time (min)	Sensitivity
Reported method [7]	C_18_ column (250 mm × 4.6 mm, 5 *μ*m) At ambient temperature	Ammonium acetate : acetonitrile (pH 3.5) (65 : 35,* v/v*)	8 min	CANA (5–30 *μ*g·mL^−1^) MET (50–300 *μ*g·mL^−1^)

Reported method [8]	C_18_ column (250 mm × 4.6 mm, 5 *μ*m) At Temperature at 30°C	Phosphate buffer : acetonitrile (pH 4.5) (65 : 35, *v/v*)	4 min	CANA (5–30 *μ*g·mL^−1^) MET (50–300 *μ*g·mL^−1^)

Reported method [9]	ODS column (250 mm × 4.6 mm, 5*µ*m) At Temperature at 30°C	Phosphate buffer : acetonitrile : methanol (pH 4.5) (40 : 40 : 20, *v/v*)	7 min	CANA (5–30 *μ*g·mL^−1^) MET (50–300 *μ*g·mL^−1^)

Proposed method (A)	C_18_ column (100 mm × 2.1 mm, 3 *μ*m) At ambient temperature	Methanol : phosphate buffer (pH 3.2) (75 : 25, *v/v*)	1.1 min	CANA (1–50 *μ*g·mL^−1^) MET (0.5–100 *μ*g·mL^−1^)

Proposed method (B)	Hypersil gold (50 mm × 2.1 mm, 1.9 *μ*m) At ambient temperature	Methanol : phosphate buffer (pH 3.5) (80 : 20 *v/v*)	1 min	CANA (0.1–50 *μ*g·mL^−1^) MET (0.25–100 *μ*g·mL^−1^)

**Table 2:** Results obtained by the HPLC-UV and UPLC-UV methods for the simultaneous determination of CANA and MET in their binary mixture.

Parameters	HPLC		UPLC	
	CANA	MET	CANA	MET
Linearity range (*µ*g·mL^−1^)	1–50	0.5–90	0.1–50	0.25–100
Slope	0.9102	0.9319	0.8821	1.0795
Intercept	0.3367	0.255	0.1499	0.5372
Correlation coefficient	0.9999	0.9999	0.9999	0.9999
Accuracy (mean ± SD)	99.81 ± 0.73	99.37 ± 0.54	99.47 ± 1.034	99.73 ± 0.89
LOD	0.7154	0.9327	0.463	0.614
LOQ	2.1075	2.8263	1.406	1.862
Precision (% RSD) Repeatability	1.31	1.47	0.48	0.71
Interday precision (reproducibility)	1.75	0.90	0.89	1.85
Specificity (mean ± SD)	99.60 ± 0.33	100.38 ± 1.20	99.89 ± 1.39	99.09 ± 1.31

**Table 3:** Determination of CANA and MET in lab prepared mixtures by the proposed methods.

Mix. number	CANA	MET	CANA		MET	
	Taken (*µ*g/mL)		Found (*µ*g/mL)	% recovery	Found (*µ*g/mL)	% recovery
HPLC method						
1	8	70	8.03	100.45	69.74	99.63
2	10	65	10.16	101.75	64.38	99.05
3	20	80	19.81	98.99	79.51	99.39
4	25	65	24.88	99.53	65.27	100.42
5^**∗**^	3	60	2.94	98.09	59.71	99.51

	*Mean ± SD*		99.76 ± 1.40		99.60 ± 0.51	

UPLC method						
1	8	70	7.94	99.21	70.20	100.29
2	10	65	9.92	99.17	65.58	100.89
3	20	80	19.85	99.23	78.64	98.29
4	25	65	24.75	99.01	65.42	100.64
5^**∗**^	3	60	3.01	100.19	59.69	99.48

	*Mean ± SD*		99.36 ± 0.47		99.92 ± 1.05	

^*∗*^Laboratory mixture represents the CANA: MET ratio as 1 : 20 as in dosage form.

**Table 4:** Assay of canagliflozin and metformin in Invokamet tablets and application of the standard addition technique.

	HPLC										UPLC									
Pharmaceutical formulation	CANA					MET					CANA					MET				
	Claimed *μ*g·mL^−1^	% found	Standard addition			Claimed *μ*g·mL^−1^	% found	Standard addition			Claimed *µ*g·mL^−1^	% found	Standard addition			Claimed *µ*g·mL^−1^	% found	Standard addition		
			Pure added	Pure found	% recovery			Pure added	Pure found	% recovery			Pure added	Pure found	% recovery			Pure added	Pure found	% recovery
Invokamet 50 CANA/1000 MET	3 *μ*g·mL^−1^	100.46	7	6.96	99.56	60 *μ*g·mL^−1^	100.15	5	4.95	99.09	3 *μ*g·mL^−1^	99.46	7	7.09	101.38	60 *µ*g·mL^−1^	98.86	5	5.01	100.22
			22	21.81	99.16			5	5.11	101.94			22	21.59	98.12			5	4.86	97.25
			17	16.96	99.82			20	19.99	99.93			17	16.93	99.58			20	19.84	99.19
			5	4.99	99.87			10	10.06	100.57			5	5.02	100.49			10	9.97	99.72

			*Mean *±* SD*		99.60 ± 0.33			*Mean *±* SD*		100.38 ± 1.20			*Mean *±* SD*		99.89 ± 1.39			*Mean *± *SD*	99.09 ± 1.31	

**Table 5:** System suitability tests for LC/UV methods for simultaneous determination of CANA and MET.

Parameters	HPLC		UPLC	
	CANA	MET	CANA	MET
Retention time (*t*_*R*_)	0.9	0.5	1	0.5
Resolution (Rs)	2.8		2.5	
Selectivity factor (*α*)	0.5		0.5	
Capacity factor (*K*′)	14	7	13	8
Number of theoretical plates (*N*)	1296	400	1600	400

**Table 6:** Statistical comparison between the results of the proposed methods and the reference methods for CANA [7] and MET [27].

Values	Proposed methods				Reported methods	
	HPLC		UPLC		CANA	MET
	CANA	MET	CANA	MET		
Mean	99.81	99.37	99.47	99.73	99.52	100.59
SD	0.73	0.54	1.034	0.89	0.76	0.81
% RSD	0.73	0.54	1.039	0.89	0.76	0.81
*n*	5	5	5	5	5	6
Variance	0.53	0.29	1.07	0.79	0.58	0.66
Student's *t*-test^b^ (2.571)	0.6154	2.866	0.08712	1.678		
*F*-value^b^ (6.388)	1.084	2.250	1.851	1.207		

^b^The values in the parenthesis are the corresponding theoretical values of *t* and *F* at *P* = 0.05.
